# Cocaine Again

**Published:** 1891-04

**Authors:** 


### Cocaine Again.

On the 2d of December, 1890, M. Hallopeau, reported to the Academie de Medecine a case of chronic cocainism induced by a single injection into the gum of 8 centigrams of hydrochlorate of cocaine. From a study of this case, he believes himself authorized to deduce the following conclusions:

A single injection of cocaine, even in a small dose, may not only produce immediate toxic symptoms of a grave character, but may give rise to symptoms persisting for several months. These distant symptoms are analogous to those perceived sometimes immediately after the injection, viz.: obstinate headache, insomnia, numbness of the extremities, attacks of faintness, dizziness, prostration, loquacity and a state of great agitation. These accidents are chiefly in very excitable subjects.

In the current number of *La Medecine Moderne*, M. Reclus, who employs cocaine largely, endeavors to controvert these statements of M. Hallopeau, and asserts that, properly managed, this valuable anæsthetic is innocuous. The rules to be followed in the management of this drug are, according to M. Reclus, as follows;

1. The quantity of cocaine injected should never exceed 12 centigrams, 2, 4, 6, or exceptionally 8 centigrams sufficing for most minor operations.

2. Employ a *weak solution* ( 2 per cent ).

3. Avoid the introduction of the drug into the interior of a blood-vessel. The best way to avoid the evil consequences of such a *contretemps* is to push the needle into the tissue slowly, and while so doing to press on the piston-rod at the same rate. In this manner, even if a vessel be pierced, only a small proportion of the solution can mingle with the blood contained in the wounded vessel.—*Paris letter to Jour. Am. Med. Ass'n.*

WASTE AND REPAIR. The object to be attained also determines our choice of diet. There are conditions of mal-nutrition in which we aim to store up food in the body. In these conditions we study the assimilation, and we use food which is easily digested. We put the individual at rest so that all his nerve force may be reserved for nutritive processes. By massage and electricity we stimulate the assimilative power and then we crowd the stomach and liver. In such conditions of mal-nutrition this crowding can be borne for a time and good results follow. Finally, however, we come to a point where the needs of the system have been satisfied, and further continuance of the sick-room diet results only in choking of the excretory channels. The stuffing process is a remedial measure. Normal working diet is altogether a different thing. A good working diet is that which supplies the body with nutritive material, little by little, as required. The food which supplies this properly is always comparatively indigestible. In the case of the laboring man it is pork and beans, hard boiled eggs, crisp, fried steak, and the like. A broiled tenderloin would not answer his purpose half so well; while for you it may be essential on account of the weakness of your digestive powers. To repeat something we have said before the stomach is like the coal-box of the engine. To empty the coal-box all at once into the furnace is neither effective nor economical. The result would be one great fire which would carry you part of the distance, and would then leave you standing on the track; while if you had supplied the same coal to the furnace by the shovelfull it would have furnished force sufficient to have completed your journey. In another respect, too, the analogy is good. The over-heated engine is often permanently damaged. The body flooded with easily digested but unassimilated foods, soon shows evidences of injury in gouty joints, diseased kidneys and liver. *The Nightingale.*

